# Chenopodium Anthelminticum

**Published:** 1887-08

**Authors:** C. B. Maclay

**Affiliations:** Peoria, Ill.


### CHENOPODIUM ANTHELMINTICUM.

BY C. B. MACLAY, M.D., PEORIA, ILL.

Under the above title we recognize an old friend, and as in this day of reputed progress old friends are hastily gotten rid of for the sake of scraping an acquaintance with some pretentious dude, so this once famous medicine is almost relegated to the shades, while new preparations "that please the children," have taken its place; and yet for the expulsion of the round worm it has no superior, nor, in fact, equal. Unfortunately, the taste of the oil is not delicious, but may be rendered very much less disagreeable by various methods of combination. The old custom was to drop it upon sugar—say from eight to ten drops on lump sugar as one dose, and repeated nightly.

It may, however, be given in small doses of castor oil or glycerin—say one-third of a teaspoonful with a few drops of essence of menth. pip. to conceal the offensive flavor. When dropped on sugar, it is well to add a drop or two of the menth. pip. ess. after the oil has been added to the sugar.

In cases where children are particularly hard to manage, it is well to give a little sugar and peppermint as the first installment, and the sugar and chenpodium oil while the mint still tickles the palate.

There can be no doubt that many of the pains and distresses of childhood are produced by the presence of the round worm in the bowels, stomach, or even esophagus, as they have been ejected from the mouth, and even from the nostrils. Cases are reported of complete strangulation caused by their forming a ball and occluding the larynx.

The round worm is sometimes present in the bowels of adults, especially females, and the reason therefor seems to be a debilitated condition of the general system, though it may be true, as alleged by some practitioners, that the presence of the worm gives rise to the sign of debility, from the annoyance to the nerves of the viscera, creating ravenous appetite and subsequent disgust for food. There is no doubt that reciprocal annoyance from ovaries to bowels, and vice versa, is often occuring in maidens, and a very reasonable belief it is that many menstrual troubles may be aided and abetted by agency of the same kind. My friend, Dr. Harris, of Groveland, tells of a "hardy" doctor who formerly made the Mackinaw woods echo with profanity, who, when called to see a youthful patient, invariably rendered his diagnosis "—— —— worms," and thereby made for himself a reputation that lingers in the "timber" to this hour.

It is said "a miss is as good as a mile," but the converse is true—"a good guess is equal to a demonstration."

Let us not forget the old remedy, "Chenopodium" or "Goose Foot."—*Peoria Medical Monthly.*

